# Optimization of high endoglucanase yields production from polypore fungus, *Microporus xanthopus* strain KA038 under solid-state fermentation using green tea waste

**DOI:** 10.1242/bio.047183

**Published:** 2019-11-29

**Authors:** Kim Anh Nguyen, Jaturong Kumla, Nakarin Suwannarach, Watsana Penkhrue, Saisamorn Lumyong

**Affiliations:** 1 Department of Biology, Faculty of Science, Chiang Mai University, Chiang Mai 50200, Thailand; 2 Master’s Degree Program in Applied Microbiology, Faculty of Science, Chiang Mai University, Chiang Mai 50200, Thailand; 3 Center of Excellence in Microbial Diversity and Sustainable Utilization, Faculty of Science, Chiang Mai University, Chiang Mai 50200, Thailand; 4 School of Preclinic, Institute of Science, Suranaree University of Technology, Nakhon Ratchasima 30000, Thailand; 5 Academy of Science, The Royal Society of Thailand, Dusit, Bangkok 10300, Thailand

**Keywords:** Agricultural wastes, Cellulolytic enzymes, Polypore fungi

## Abstract

Polypores are diverse macrofungi that have been extensively studied for their lignocellulolytic enzyme production capabilities. Currently, these enzymes are being used for many industrial purposes. However, the high cost associated with their production is the main barrier to their broader application. This work aimed to study the optimal medium and conditions for endoglucanase production using solid state fermentation. Seven polypore strains were used for endoglucanase activity screening. The fermentation experiments were carried out in 250 ml Erlenmeyer flasks with green tea waste as a substrate. Notably, *Microporus xanthopus* strain KA038 showed the best level of activity (38.62 IU/gds). Various parameters such as moisture content, nitrogen source, initial pH value, inoculum size and incubation time were considered to determine the optimal conditions for endoglucanase production. The optimal medium consisted of green tea leaves as a carbon source, beef extract as an organic nitrogen source, NH_4_H_2_PO_4_ as an inorganic nitrogen source, pH 7.0 and an incubation temperature at 30°C for 4 days resulted in a high enzyme yield with *M. xanthopus* strain KA038 (81.8 IU/gds).

This article has an associated First Person interview with the first author of the paper.

## Abstract (precis)

**Summary:** Our findings show that one polypore fungal species *Microporus xanthopus* strain, KA038, can produce high endoglucanase yields through optimizing production conditions.

## INTRODUCTION

Lignocellulosic biomass is mainly formed of three polymers; cellulose, hemicellulose and lignin along with other components (Isikgor and Becer, 2015). Despite the usefulness of these materials in many sectors of the economy, a great amount of the lignocellulosic biomass ends up being discarded and becomes a pollutant (Vintila et al., 2009). On the other hand, lignocellulose biomass is a source material that has been used in previous decades in the production of bioethanol, organic acids, enzymes and biodegradable plastics in the form of cheap carbohydrates (Ravindran and Jaiswal, 2016). Lignocellulosic material is promising as an energy source because of its potential for low-cost fermentation (Sherief et al., 2010). Green tea is considered one of the most popular drinks in the world and the custom of drinking tea has spread quickly and widely throughout the world (Kondo et al., 2004). According to Kirci (2006) and Tutus et al. (2014), green tea residues are composed of 26.53±0.58% alpha cellulose, 29.42±0.57% cellulose, 60.81±1.14% holocellulose and 36.94±0.34% lignin. However, a substantial amount of tea waste is left after the tea extraction process. Thus, looking elsewhere to minimize or eliminate tea waste has been a difficult task in the tea processing industry (Yang et al., 2015). Hence, at present, the elimination of tea waste in the environment in an economically sound and efficient manner has become of significant interest among scientists.

In contrast with submerged fermentation (SmF), solid state fermentation (SSF) is a procedure wherein the substrate is fermented and barely appears in water. The low moisture content in the fermentation of microorganisms is limited primarily to yeasts, fungi and bacteria (Acharya et al., 2010). One of the most adaptive organisms to SSF is fungi because the fugal hyphae features are spread across the surface and easily penetrate inter particle spaces, thereby their colonization is typically more efficient than other organisms in solid substrates (Sukumaran et al., 2005).

Polypores (basidiomycetes) are a group of wood-decaying fungi that are diverse in ecological specificity and morphological characteristics. Most polypores are capable of breaking down lignocellulose, and consequently they play a mainstay role in nutrient recycling in forest ecosystems. Although some polypores can cause tree diseases such as *Onnia tomentosa*, *Phaeolus schweinitzii* and *Phellinidium weirii* (Ginns, 2017), others have medicinal properties (Jayachandran et al., 2017). Notably, the ability of polypores to produce enzymes has attracted the attention of scientists. In recent years, researchers have become interested in the screening and production of cellulolytic enzymes from polypores (Table 1), and the resulting research has shown positive results. Zecarias et al. (2016) screened ligninocellulolytic enzymes by the dye decolorization plate test, with 49 of 61 fungal strains showing decolorizing activity on Phenol Red, Azure B and RBBR agar. The exoglucanase and β-glucosidase production were induced using the co-culture between *Trichoderma viride* and *Ganoderma lucidum* in SSF (Shahzadi et al., 2014). Cellulase is an enzyme that converts cellulose into simple sugars (glucose) (Chinedu et al., 2005). Many studies have reported that microorganisms and animals are able to hydrolyze β-1,4 linkages in cellulose (Henrissat, 1991). The production rate of cellulase obtained from fungi is higher than from other microorganisms and this can be advantageous (Rana and Kaur, 2012). Generally, complete cellulose hydrolysis is responded to by three main types of cellulase combinations including endoglucanases, exoglucanases and β-glucosidase (Zhang and Lynd, 2006). These enzymes are widely used in numerous application areas including the beverage, paper and textile industries, in agriculture, detergents and animal feeds, as well as serving as an important alternative source of energy generation. Some organic compounds are inducers for cellulase such as disaccharides, spent ammonium sulphite liquor (Han et al., 2017) and glycerol (da Silva Delabona et al., 2016). This research study aims to explore the isolation of polypore fungi and the production of endoglucanase enzyme.

**Table 1.**
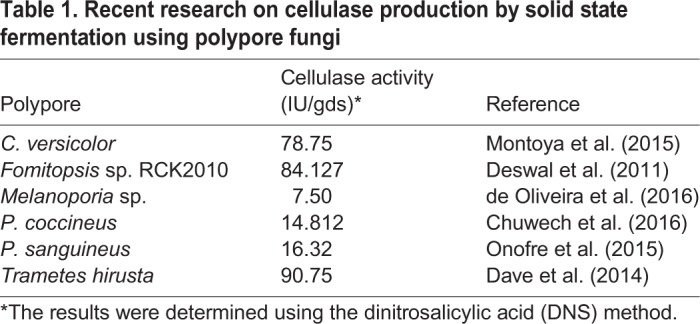
**Recent research on cellulase production by solid state fermentation using polypore fungi**

## RESULTS

### Fungal identification

**Fig. 1.**
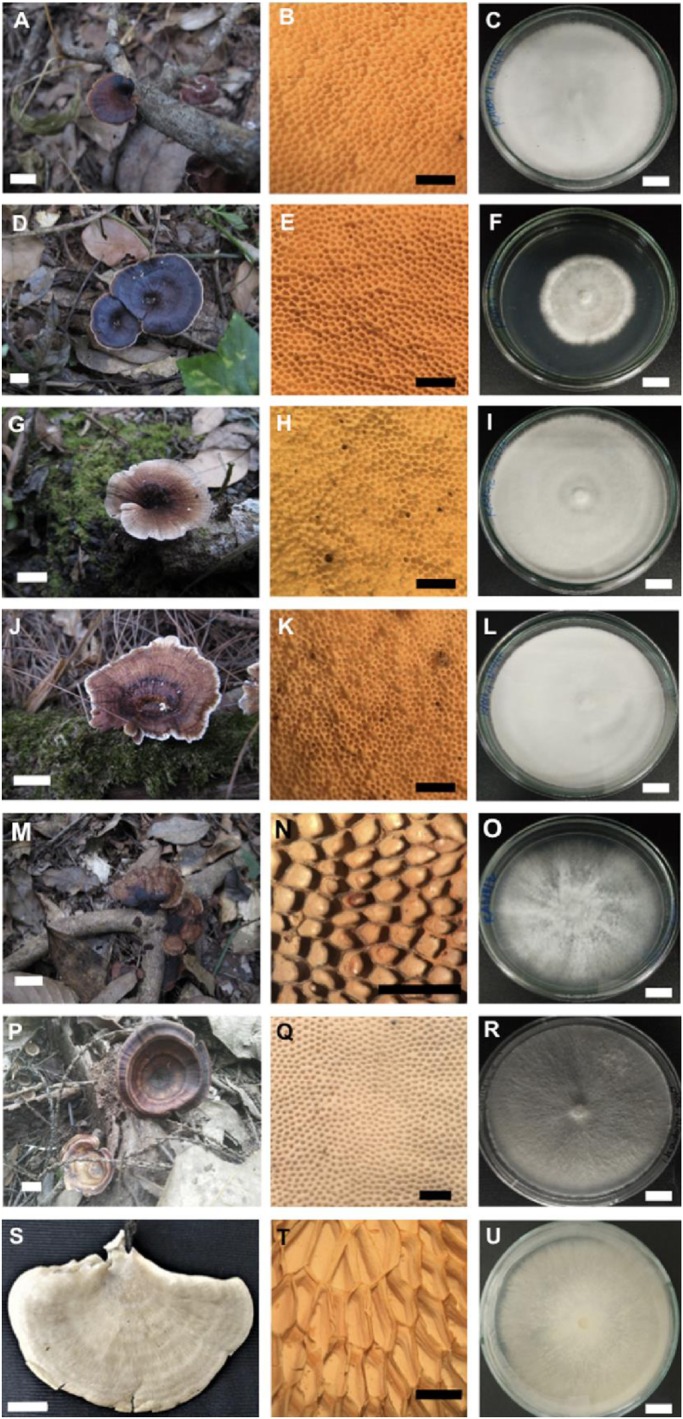
**Fruiting body, pores and** **7-day-old colonies** **on PDA media of polypore fungi used in this study.** (A–C) *Microporus affinis* (KA007), (D–F) *M. affinis* (KA009), (G–I) *M. affinis* (KA012), (J–L) *M. affinis* (KA016), (M–O) *H. tenuis* (KA018), (P–R) *M. xanthopus* (KA038), (S–U) *Favolus* sp. (KA053). Scale bars: A,C,D,F,G,I,J,M,N,O,P,S,U: 1 cm; B,E,H,K,N,Q: 20 µm; T: 40 µm.

In this study, the polypore samples were collected based on their distinct morphology. A total of seven strains were selected from dead logs (Fig. 1).

Based on the morphological characteristics, five strains (KA007, KA009, KA012, KA016 and KA038) were classified into the genus *Microporus*. The strain KA018 and KA053 were classified into the genus *Hexagonia* and *Favolus*, respectively. The molecular results were used to confirm the fungal identification.

**Fig. 2.**
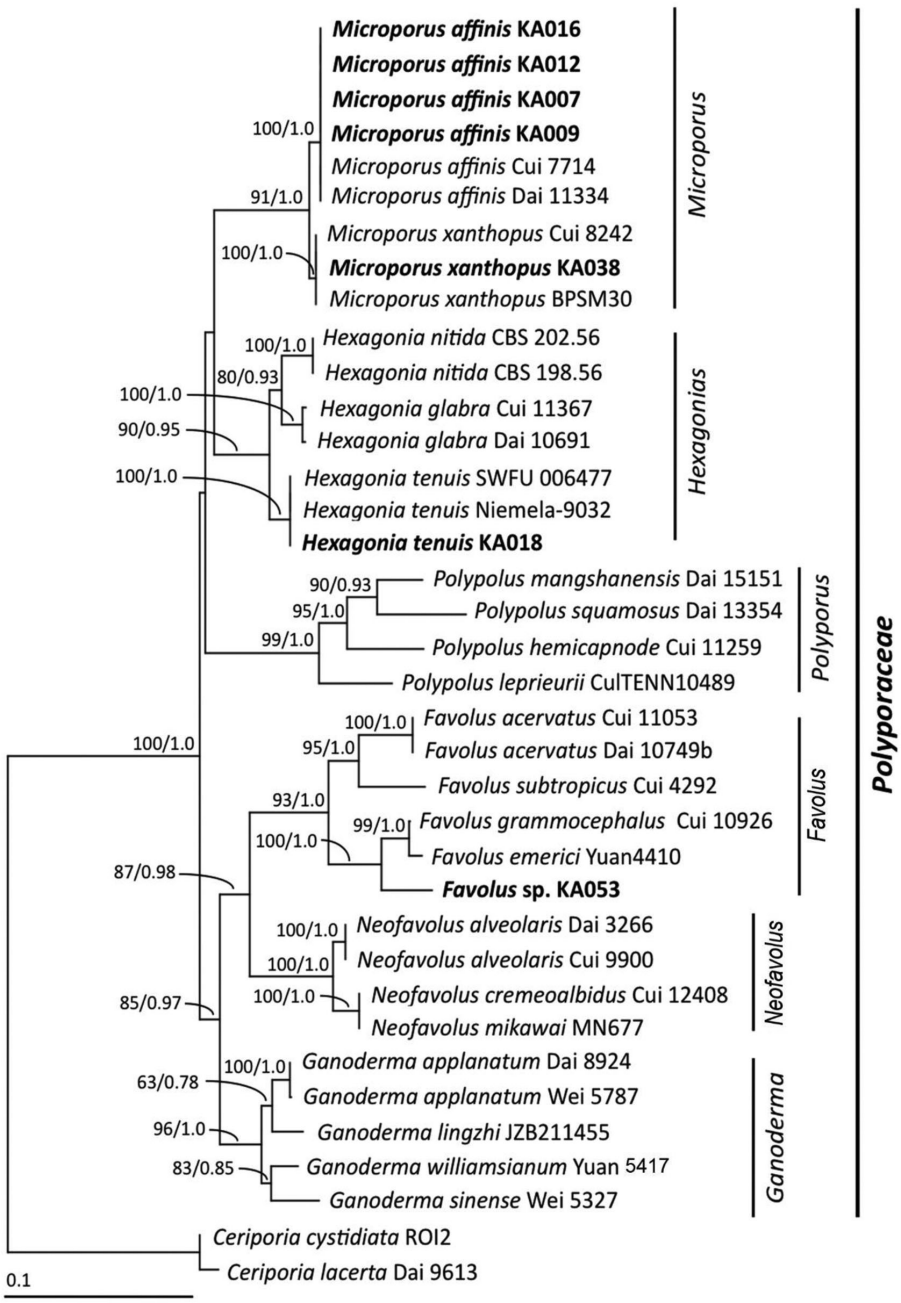
**Phylogenetic tree derived from maximum likelihood analysis of combined ITS, LSU and *rpb1* genes of 37 sequences.** *Ceriporia cystidiata* and *C*. *lacerate* were used as outgroup. Numbers above branches are the bootstrap statistics percentages (left) and Bayesian posterior probabilities (right). Branches with bootstrap values ≥50% are shown at each branch and the bar represents 0.1 substitutions per nucleotide position. The fungal isolates from this study are in bold.

A phylogram indicated that all fungal isolates in this study were placed within the family Polyporaceae (Fig. 2). Five fungal strains were placed into the genus *Microporus*, within which the strains KA007, KA009, KA012 and KA016 were identified as *Microporus* *affinis* and the strain KA038 was *Microporus* *xanthopus*. Additionally, the fungal strain KA018 was identified as *Hexagonia tenuis*. The remaining fungal strain, KA053, was placed in the genus *Favolus*, which forms a sister taxon to *Favolus* *grammocephalus* and *Favolus* *emerici*.

### Screening of endoglucanase production ability

#### First screening: plate screening using carboxymethyl cellulose (CMC) as carbon source

**Fig. 3.**
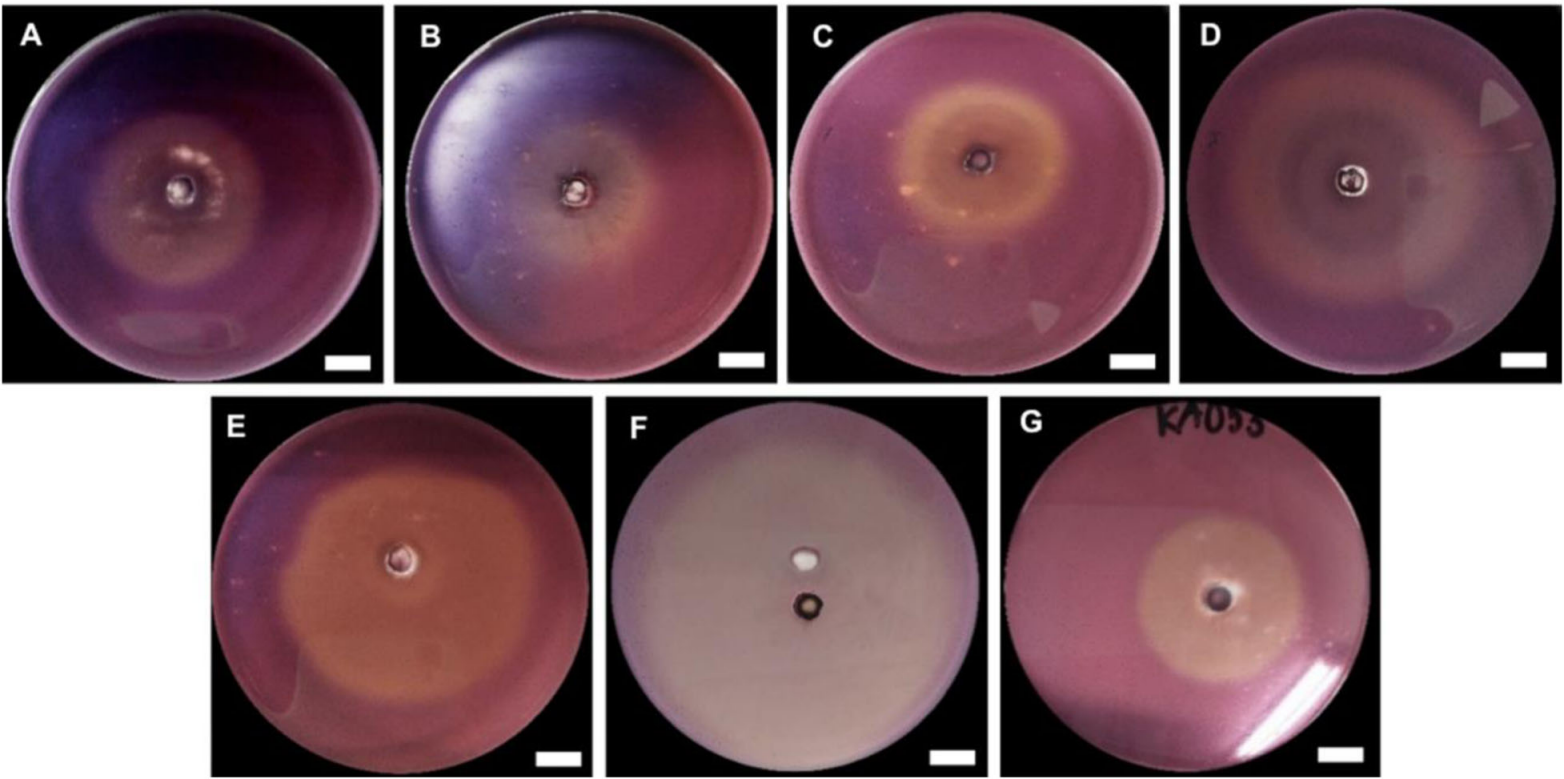
**Observation of the clear zone around a colony of polypores using Congo Red dye.** Scale bars: 1 cm.

The first screening of endoglucanase production was performed using the Congo Red test. This test was based on the measurement of colony growth and hydrolysis halos were used for enzymatic index (EI) calculation. The production of the halos by cellulose hydrolysis was directly related to the region of action of cellulolytic enzymes, as the stain only remains attached to β-1,4-D-glucanohydrolase bonds (Lamb and Loy, 2005). The seven fungal strains showed the halo zone on CMC agar. The results showed that the strain KA016 showed the highest degree of enzymatic index at 1.5 (Fig. 3 and Table 2).

**Table 2.**
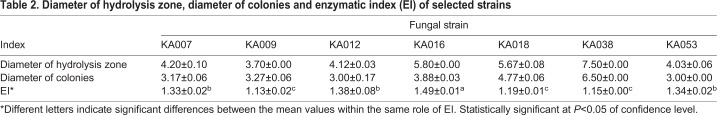
**Diameter of hydrolysis zone, diameter of colonies and enzymatic index (EI) of selected strains**

#### Second screening: screening of endoglucanase production ability in liquid medium

**Fig. 4.**
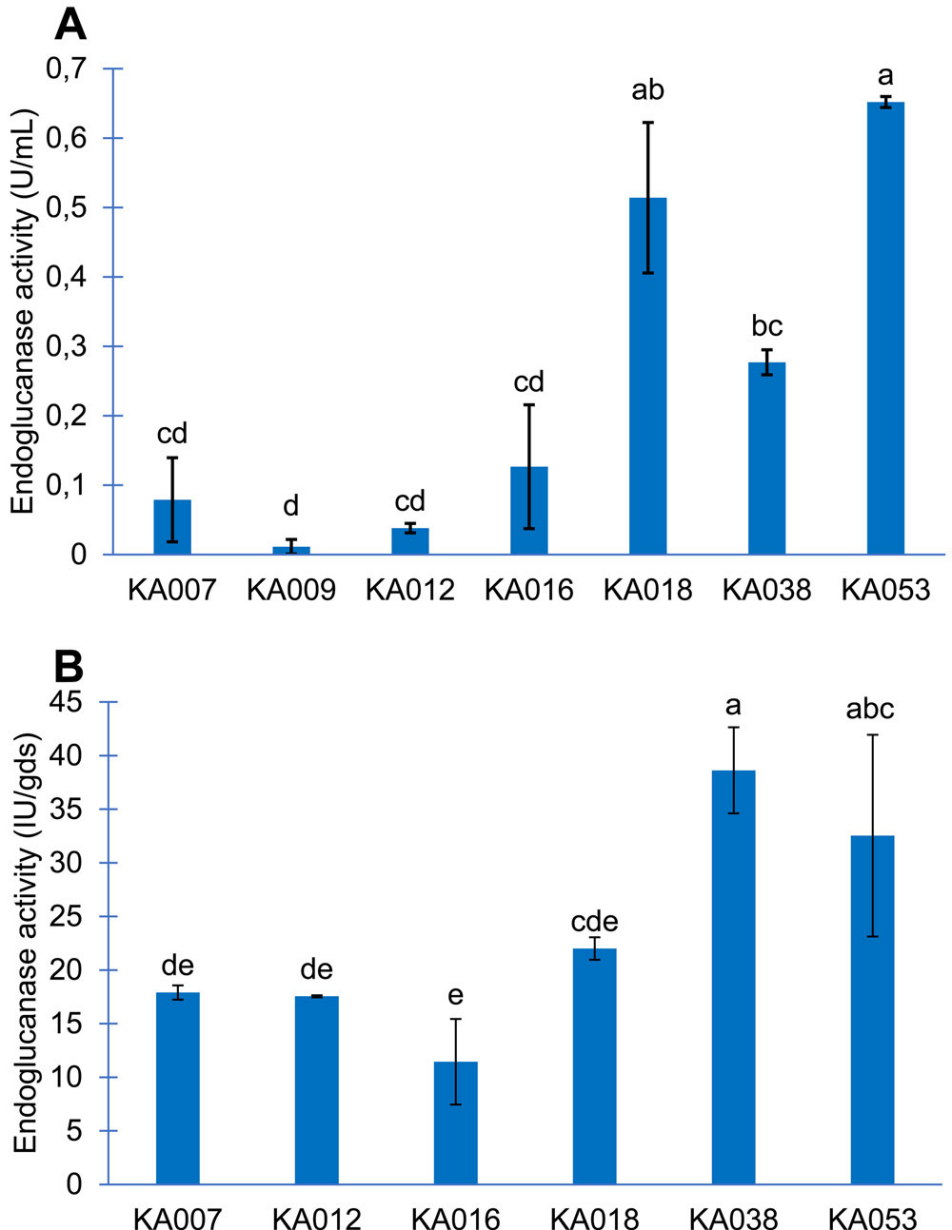
**Screening and evaluation of potential endoglucanase activity producing polypore strains using liquid media by DNS assay (A).** Endoglucanase activity by polypore fungi on green tea waste substrate by DNS assay (B). The different letters in each graph indicate a significant difference.

All strains were positive for endoglucanase production and were able to grow in CMC agar, but the strains differed in their ability to produce endoglucanase (Table 3 and Fig. 4A). The strain KA016 revealed the highest EI value (1.49) in agar diffusion method, the strain KA053 revealed the highest endoglucanase activity (0.65 U/ml) in liquid fermentation, whereas KA009 indicated the smallest clear zone diameter (EI 1.12) and the lowest endoglucanase activity (0.01 U/ml). As a result, only strain KA009 was not considered for further studies.

**Table 3.**
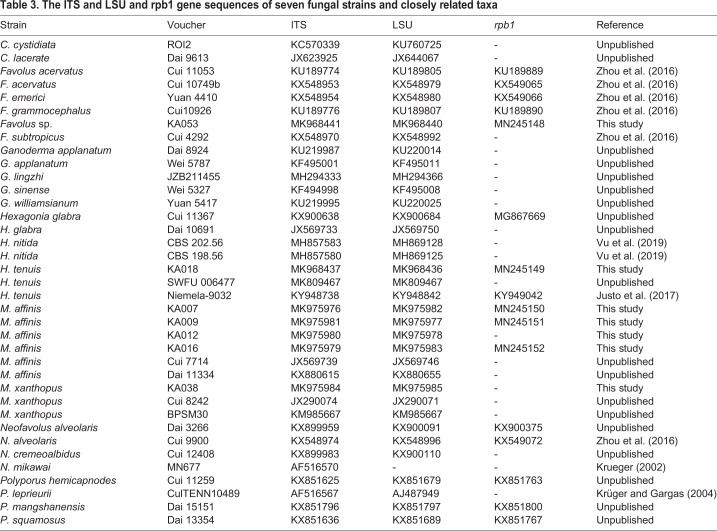
**The ITS and LSU and rpb1 gene sequences of seven fungal strains and closely related taxa**

#### Third screening: screening on substrate by solid state fermentation

The different degrees of potential to produce endoglucanase of various strains was studied by testing the activity of enzyme production using green tea as the substrate. The highest enzyme activity was observed in strain *M. xanthopus* strain KA038 (38.62 IU/gds) (Fig. 4B). Consequently, only KA038 was selected for further experiments.

### Optimization of conditions for endoglucanase production by *M. xanthopus* strain KA038 in solid state fermentation

#### Effect of moisture content

**Fig. 5.**
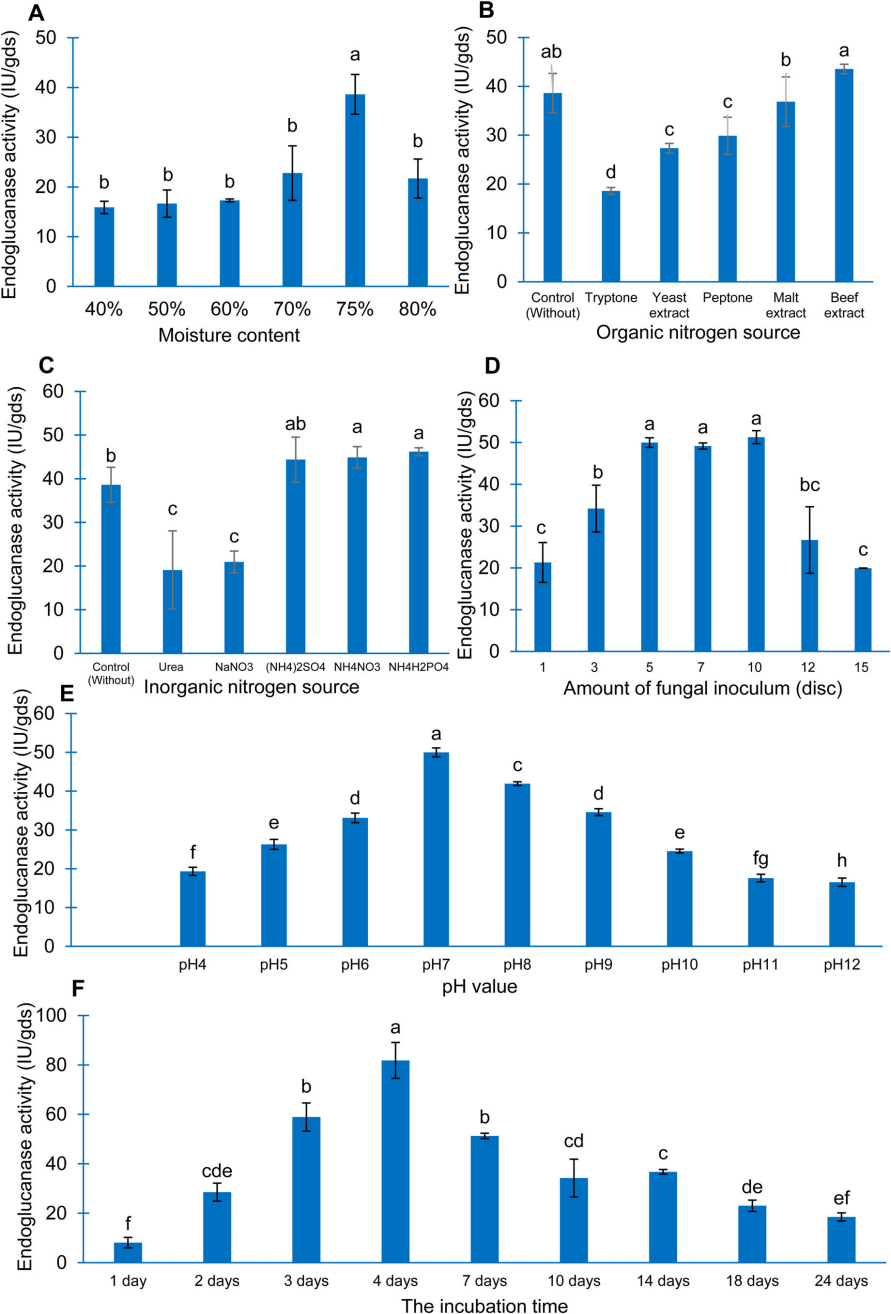
**Effect of conditions for endoglucanase** **production by *M.*** ***xanthopus* strain KA038.** (A) Effect of moisture content. (B) Effect of organic nitrogen source. (C) Effect of inorganic nitrogen source. (D) Effect of the amount of fungal inoculum. (E) Effect of pH value. (F) Effect of incubation time. The different letters in each graph indicate a significant difference.

For the *M. xanthopus* strain KA038, the optimal moisture content was 75% and activity was recorded at 38.62 IU/gds (Fig. 5A). The lower or higher moisture content in the solid state fermentation did not help to increase endoglucanase activity. The results indicated that only at 75% of moisture content lead to an increase in endoglucanase activity.

#### Effect of nitrogen source

The result of the effect of various nitrogen sources on endoglucanase production is illustrated in Fig. 5B and C. Among the different nitrogen sources, the highest degrees of endoglucanase production were 43.5 IU/gds and 46 IU/gds, when beef extract was added as an organic nitrogen source and NH_4_H_2_PO_4_ was added as a source of inorganic nitrogen, respectively.

#### Effect of pH value

The effect of pH on endoglucanase production was determined at pH values ranging from 4.0–12.0 and shown in (Fig. 5E). Green tea waste revealed a maximum endoglucanase activity of 49.9 IU/gds, which was observed at pH 7.0. There was drastic dropped in endoglucanase activity from pH 8.0 onward.

#### Effect of the amount of fungal inoculum

Maximum activity was noted with ten discs of inoculum amount of the inoculated strain (Fig. 5D). By increasing the inoculum amount, an increase in enzyme activity was recorded. The maximum endoglucanase activity recorded was 51.27 IU/gds with ten discs of inoculum. The results from five, seven and ten discs of fungal inoculum were not significantly different, statistically, however, using ten discs of fungal inoculum showed the highest potential to produce endoglucanase (51.27 IU/gds), whereas five and seven discs of fungal inoculum showed 49.97 and 49.14 IU/gds, respectively. In this research, optimal conditions were a priority.

#### Effect of incubation time

The results revealed that the maximum endoglucanase activity (81.8 IU/gds) was observed at an incubation time of 4 days for *M. xanthopus* strain KA038. The enzyme activity gradually increased with a decrease in incubation time up to the optimum level, followed by a gradual fall in activity (Fig. 5F).

## DISCUSSION

The examination of endoglucanase activity was carried out by staining the isolates of the fungus with Congo Red. This method can definitely be applied for screening endoglucanase producing fungal strains. In this research, all the isolates – *Favolus* sp. strain KA053, *H.* *tenuis* strain KA018, *M.* *xanthopus* strain KA038, *M. affinis* strain KA007, *M. affinis* strain KA009, *M. affinis* strain KA012 and *M. affinis* strain KA016 – showed a positive zone of clearance on screening. In Congo Red staining, the enzymatic halo changed from red to opaque orange. Accordingly, the Congo Red method can be used as a rapid test, a sensitive reaction for screening endoglucanase-producing fungi. However, dye screening methods are not a quantitative method for analyzing enzyme activity through halo size. This problem was overcome by using short cello-oligosaccharides that modified the chromogenic/fluorogenic groups, e.g. fluorescein, resorufin and 4-methylumbelliferone, leading to higher sensitivity and quantification (Fia et al., 2005). Enzyme activity of produced endoglucanase without substrates was measured in units/ml. One unit was designated as the quantity of endoglucanase enzyme released 1 μmol of reducing sugars commensurate to glucose per 1 min during the reaction. In this study, some strains showed big and clear halo zones (high EI), but their potential to produce endoglucanase that could be measured by a secondary screening was inconsistent, demonstrating that either the enzyme concentration producing these strains was lower or higher after cultivation in the liquid medium, or that the potential of the strains to excrete endoglucanase was not good. Sadhu and Kanti have shown that the hydrolyzing zone diameter may not be the direction of the real cellulolytic enzyme activity (Sadhu and Kanti, 2013).

Moisture is an essential factor in the SSF process. Consequently, this adjustment has a significant influence on growth, microbe biosynthesis and secretion of metabolites such as enzymes. It has been reported that the production of enzymes was negatively affected in instances of increased or decreased moisture. Ahmed (2008) and Sodhi et al. (2005) claimed that the crucial factor influencing the outcome of SSF is the moisture content of the substrate, and that the reduction of nutrients provided to the organisms resulted in lower moisture content. On the other hand, high moisture caused a reduction in enzyme production that may have been due to the substrate porosity reduction; the structure of substrate particles changed, the gas volume decreased and microbial growth was reduced (Baysal et al., 2003). This result was achieved in an upscale screening on the substrate by solid state fermentation, and a better result was observed than with other moisture content levels. The optimal moisture content of 75% was used for further experiments.

The nitrogen source plays a role as a secondary energy source for the organisms; they are important for producing industrial fermentation mediums that are designed to ensure maximum enzyme production. In this experiment, different inorganic and organic nitrogen sources, which have an influence on the level of fungal growth and enzyme production, were screened at 0.1% concentration. Our results indicate that the nitrogen source strongly affected endoglucanase enzyme production. The results show that good endoglucanase production can be obtained with both organic nitrogen sources (such as beef extract, malt extract and ammonium compound) and inorganic nitrogen sources [such as NH_4_H_2_PO_4_, NH_4_NO_3_ and (NH_4_)_2_SO_4_]. But enzyme production was remarkably decreased in the presence of tryptone, yeast extract, peptone, urea and NaNO_3_. These are in accordance with submitted papers that have presented the idea that ammonium compounds are the most prosperous nitrogen sources for endoglucanase synthesis. However, even though the addition of organic nitrogen sources such as beef extract and malt extract resulted in increased enzyme production – as was reported before – because of their higher cost they were not an effective replacement for inorganic nitrogen sources (Tao et al., 1999).

Regarding the physical parameters, the pH of the growth medium plays an important role by inducing morphological changes in polypore fungi and in enzyme secretion. Every enzyme shows maximum activity at a certain pH. There has been much work that attempts to achieve the maximum cellulolytic enzyme productivity by optimizing pH value (Li et al., 2013; Teixeira Da Silva et al., 2016). The enzyme was still active over a wide pH range and it was observed that the endoglucanase activity had a broad pH range of between 4.0–12.0. This was due to the fact that cultivation of fungi at an unfavorable pH value may result in reduced enzyme activity by reducing the accessibility of the substrate (Bakri et al., 2008). In the SSF process, for optimizing the initial pH precautionary measures were taken due to the fact that extracellular enzymes were only stable at a particular pHs, and there may be rapid denaturation at lower or higher values. Notably, the metabolic activities of the microorganism are very sensitive to changes in pH because pH affects the cellulolytic enzyme production of fungi (Kalra and Sandhu, 1986).

A lower amount of inoculum requires a longer time for the colonies to multiply to a sufficient number to utilize the substrate and produce the enzyme. An increase in the number of colonies in the inoculums would ensure rapid proliferation and biomass synthesis. Finding a balance between the increasing biomass and accessible nutrients would yield optimal enzyme production (Ramachandran et al., 2004). Higher inoculum size might accelerate the fungal growth rate but at the same time increase the rate of nutrient depletion. Upon nutrient depletion the growth of the fungi is affected and this might not be helpful in improving the yield of cellulolytic enzyme production (Kumaran et al., 1997). In this study, endoglucanase production increased together with an increase of initial inoculum size ranging from one to ten discs in inoculated strains. Maximum cellulase production (51.27 IU/gds) was observed at an inoculum of ten discs in inoculated strains. Higher inoculum levels (>ten discs) increased the colonies’ density per gram of solid substrate; this factor hindered the penetration of oxygen into the solid medium, inhibiting endoglucanase production. However, a lower inoculum size of the colonies’ culture extended the time required for the fermentation process. Notably, there is an indispensable factor influencing the optimal inoculum amount in SSF because smaller inoculations can be short of biomass and allow unexpected organism growth, whereas a high inoculation may tend to be full of biomass and deplete the necessary nutrients (Bogar et al., 2003). Irrespective of SSF or SmF, inoculum amount can greatly influence the yield of the final product (Prakasham et al., 2006).

Fermentation time has an important impact on enzyme production; enzyme production is related to incubation time (Gautam et al., 2011). The time course for the production of endoglucanase enzyme was investigated and a fermenting for a period of 1–24 days was carried out. As shown in Fig. 5, maximum endoglucanase activity was found after 4 days, where the maximum activity was 81.8 IU/gds. A further increase in the incubation period leads to a decrease in the production of endoglucanase. The decrease in enzymatic activity with time might be due to the depletion of macro- and micronutrients in the fermentation medium over time, which stressed the fungal physiology resulting in the inactivation of secreting machinery in the enzymes (Haq et al., 2010). Another reason may be the high viscosity of the medium, which decreases the oxygen supply to the microorganisms. High viscosity leads to retarded cell division, resulting in low production and excretion of cellulase (Haque and Mozaffar, 1992). This trend of decreased enzyme activity may have been due to the depletion of macro- and micronutrients in the fermentation medium over time, which stressed the fungal physiology resulting in the inactivation of the secretary machinery of the enzyme (Rajendran et al., 2015).

The potential of utilizing green tea waste as a substrate for endoglucanase production under SSF by isolated polypore fungi was identified and studied. The following were found to be the optimum SSF conditions for 5 g of substrate for *M. xanthopus* KA038: incubation at 30°C, moisture content at 75%, using beef extract and NH_4_H_2_PO_4_ as sources of nitrogen and an optimum pH of 7.0. The incubation time of 4 days produced the best endoglucanase activity, which increased from 51.27 IU/gds to 81.8 IU/gds. In this study, endoglucanase activity increased by 1.6-fold after a one factor at a time optimization procedure was employed using *M. xanthopus* KA038. The previous research using green tea wastes for solid state fermentation for cellulase production by *Trichoderma reesei* was 6.27 IU/gds (Mao et al., 2015), so our result was 13-fold times better. Compare with Ramapraba (2004); this research used *M. xanthopus* to produce cellulolytic enzymes by SmF using paddy husks as substrate and the results indicated that the highest cellulase activity (0.98 mmol/min/mg) was achieved after 6 days of incubation using 6 g of yeast extract as a nitrogen source. However, because of the higher cost, using a large amount of organic nitrogen source such as yeast extract was not the most efficient method for industrial enzyme production. Our research did not show the best result of endoglucanase production, however, it indicated the potential of polypore fungi *M. xanthopus* strain KA038 to produce better cellulolytic activity compared with other polypore fungi such as *Melanoporia* sp., *Pycnoporus sanguineus*, *Pycnoporus* *coccineus* and *Coriolus* *versicolor* (Montoya et al., 2015; Onofre et al., 2015; Chuwech et al., 2016; de Oliveira et al., 2016). Thus, this result demonstrated that *M. xanthopus* was a remarkable species of polypore fungi in producing endoglucanase activity.

## MATERIALS AND METHODS

### Fungal collection and pure culture isolation

Basidiocarps of polypore fungi were collected from dead tree stumps in Chiang Mai Province, Thailand, from December 2016 to July 2017. Tissue culture technique was employed to isolate the fungi. The inside tissues from each basidiocarp were then placed on potato dextrose agar (PDA) plate and then incubated at 30°C in an incubator. The cultures were purified by repeated transfer to fresh PDA plate every 7 days. Basidiocarps were dried at 40–45°C. The dried specimens were then deposited in the herbarium at the Sustainable Development of Biological Resources Laboratory, Chiang Mai University.

### Fungal identification

Macro-morphological characteristics of the hymeneal surface were observed using fresh specimens, based on fresh material and documented all aspects of size, shape, color and textural features (Hesler and Smith, 1979). The pertinent micro-morphological features were recorded using both stereo microscope and light microscope.

For phylogenetic analyses, the sequences from this study, previous studies and the GenBank database were used and are provided in Table 3. The multiple sequence alignment was carried out using MUSCLE (Edgar, 2004). The combined ITS, LSU and *rbp1* sequence dataset consisted of 37 taxa and were comprised of 3268 characters including gaps (ITS: 1–660, LSU: 661–2012 and *rbp1*: 2013–3268). Phylogenetic trees were constructed using maximum likelihood (ML) and Bayesian inference (BI) algorithms, implemented by RAxML v7.0.3 (Stamatakis, 2006) and MrBayes v3.2.6 (Ronquist et al., 2012), respectively. *Ceriporia cystidiata* and *C*. *lacerate* were used as outgroup. For ML analysis, the bootstrap (BS) replicates were set as 1000 and used to test phylogeny (Felsenstein, 1985). Clades with BS values of ≥70% were considered significantly supported (Hillis and Bull, 1993). For the BI analysis, the Markov chains were run for one million generations, with six chains and random starting trees. The chains were sampled every 100 generations. Among these, the first 2000 trees were discarded as burn-in, while the post-burn-in trees were used to construct the 50% majority-rule consensus phylogram with calculated Bayesian posterior probabilities. Bayesian posterior probabilities (PP)≥0.95 were considered as significantly supported (Alfaro et al., 2003).

### Screening of endoglucanase production ability

#### First screening: plate screen using CMC as carbon source

Fungal colonies (0.5 cm diameter) from 1-week-old PDA plates were cut by pasture pipette and then inoculated into the middle of the CMC agar plates. The plates were then incubated at ambient temperatures (28±2°C) for 3 days. The plates were colored with 0.1% (w/v) Congo Red for 0.5–1 h and discolored for 15–20 min with 1 M NaCl solution. Finally, 1 M NaCl was discarded and the plates were washed with water at a pH of 2.0. The staining of the plates was analyzed by observing the formation of clear or yellowish zones around the fungal colonies. Endoglucanase production ability was revealed by the size of a clear halo that was considered to be indicative of the zone of hydrolysis. This area was measured for EI calculation by dividing the diameter of the hydrolysis zone by the diameter of the colony (Béguin and Aubert, 1994).

#### Second screening: screening of endoglucanase production ability in liquid medium

Endoglucanase production ability of seven fungal strains was checked using the DNS method (Miller, 1959). Inoculum was prepared by inoculating 0.25 ml solution from 7-day-old fungal culture in PDB at 30°C with 180 rpm shaking. Supernatant was collected after 3 days of incubation and centrifuged at 10,000 rpm for 10 min at 4°C. The reaction mixture containing 0.5 ml of the sample, 0.5 ml of sodium citrate buffer and 0.5 ml of 1% (w/v) CMC was then incubated at 37°C for 60 min. The reaction was ended by adding 1 ml of DNS reagent. The color was then increased by boiling for 5 min. The measurement of optical density was performed at 540 nm (Miller, 1959).

#### Third screening: screening on substrate by solid state fermentation

Green tea waste was procured from Amazing Tea Chiang Mai Company, Chiang Mai, Thailand. It was ground using a blender. Citrate buffer was prepared by mixing sodium citrate and citric acid and the pH was adjusted to 5.0. All chemicals were of the highest reagent grade commercially available.

Endoglucanase production was studied in flasks containing 5 g of green tea waste as a substrate containing 20 ml of distilled water with moisture content at 75%. The flasks were then sterilized at 121°C for 15 min. The strains were grown on PDA plates for 7 days and added to fermentation medium as an inoculum. The flasks were incubated at 30°C for 7 days. The fermented substrate of each flask was extracted using 50 ml of 50 mM citrate buffer (pH 5.0) and filtered through the sterilized cloth in an ice bucket. The crude extract was centrifuged at 8000 rpm for 5 min, the supernatant was collected and used as a crude enzyme. The reaction mixture after that was used for the endoglucanase assay using the DNS method (Miller, 1959). The enzyme blank was also carried out in flasks containing 5 g of green tea waste containing 20 ml of distilled water. The flasks were then sterilized at 121°C for 15 min. The flasks were incubated at 30°C for 7 days. The fermented substrate of each flask was extracted using 50 ml of 50 mM citrate buffer (pH 5.0) and filtered through the sterilized cloth in an ice bucket. This crude extract was centrifuged at 8000 rpm for 5 min, the supernatant was collected and used as enzyme blank to measure the supernatant's color.

### Optimization of conditions for endoglucanase production by selected fungal strain in solid state fermentation

#### Fungal strain

The highest endoglucanase-producing strain (*M*. *xanthopus* strain KA038) was selected and used in this experiment.

#### Effect of moisture content

The moisture content optimization was determined. Three flasks containing 5 g of dried green tea waste and five discs of inoculum were adjusted to different moisture contents of 40, 50, 60, 70, 75 and 80%, adding water at 8, 10, 13, 15, 20 and 25 ml, respectively. The moisture content percentage was calculated using the subtraction of wet sample weight and dry sample weight, then divided by wet sample weight, finally multiplied with 100. The flasks were then inoculated at 30°C for 7 days. The endoglucanase activity was measured using the DNS method. The optimal moisture content was selected for further experiment.

#### Effect of nitrogen source

Five organic nitrogen sources (peptone, beef extract, yeast extract, malt extract and tryptone) and five inorganic nitrogen sources [urea, NaNO_3_, (NH_4_)_2_SO_4_, NH_4_NO_3_ and NH_4_H_2_PO_4_] at 0.1% (w/v) concentration were added into 125 ml Erlenmeyer flasks that contained 5 g of dried green tea and adjusted to optimal moisture content (75%). Five discs of fungal strain were inoculated. The flasks were incubated for 7 days at 30°C and enzyme assay was then performed using the DNS method. The optimal nitrogen source was selected for further experiment.

#### Effect of pH value

The optimized pH value was performed by adjusting the pH to 4.0, 5.0, 6.0, 7.0, 8.0, 9.0, 10.0, 11.0 and 12.0 of the optimal medium containing green tea waste using 0.1 N HCl or 0.1 N NaOH. After inoculation with five discs of inoculum, the flasks were incubated in an incubator at 30°C for 7 days and enzyme assay was then performed using the DNS method. The optimal pH was selected for further experiment.

#### Effect of the amount of fungal inoculum

The inoculum amount was optimized by preparing the inoculum on PDA plates containing fungal strain using a sterile pasture pipette of 0.5 mm in diameter. Green tea waste containing fermentation media was inoculated with one, three, five, seven, ten, 12 and 15 disc(s) of the strain aseptically. After inoculation, the flasks were incubated at 30°C for 7 days. At regular intervals, the enzyme assay was performed. The optimal inoculum dosage was selected for further experimentation.

#### Effect of incubation time

To determine the effect of incubation time on endoglucanase enzyme production, the flasks contained the optimal conditions in previous steps were inoculated with the selected strain and incubated for different numbers of days (1, 2, 3, 4, 10, 14, 18 and 24 days). The enzyme activity was determined at 540 nm using the DNS method.

### Statistical analysis

All experiments were carried out in triplicate, and all the endoglucanase activities were reported as the mean±standard deviation of three identical values. To compare the data, analysis of variance (ANOVA) was performed using SPSS 17.0. The comparison between the mean values was done using the Duncan multiple range test with a significance level of *P*<0.05. The optimization of variables carried out by one factor at a time design.

## References

[BIO047183C1] Acharya B. K., Mohana S., Jog R., Divecha J. and Madamwar D. (2010). Utilization of anaerobically treated distillery spent wash for production of cellulases under solid-state fermentation. *J. Environ. Manage.* 91, 2019-2027. 10.1016/j.jenvman.2010.05.001 20627545

[BIO047183C2] Ahmed S. A. (2008). Optimization of production and extraction parameters of *Bacillus megaterium* levansucrase using solid state fermentation. *J. Appl. Sci. Res.* 4, 1199-1204.

[BIO047183C3] Alfaro M. E., Zoller S. and Lutzoni F. (2003). Bayes or bootstrap? A simulation study comparing the performance of bayesian markov chain monte carlo sampling and bootstrapping in assessing phylogenetic confidence*. Mol. Biol. Evol.* 20, 255-266. 10.1093/molbev/msg028 12598693

[BIO047183C4] Bakri Y., Jawahar M. and Arabi M. I. E. (2008). Improvement ofxylanase production by *Cochliobus sativus* insubmerged culture. *Food Technol. Biotech.* 46, 116-118.

[BIO047183C5] Baysal Z., Uyar F. and Aytekin Ç. (2003). Solid state fermentation for production of α-amylase by a thermotolerant *Bacillus subtilis* from hot-spring water. *Proc. Biochem.* 38, 1665-1668. 10.1016/S0032-9592(02)00150-4

[BIO047183C6] Béguin P. and Aubert J.-P. (1994). The biological degradation of cellulose. *FEMS. Microbiol. Rev.* 13, 25-58. 10.1111/j.1574-6976.1994.tb00033.x 8117466

[BIO047183C8] Bogar B., Szakacs G., Linden J. C., Pandey A. and Tengerdy R. P. (2003). Optimization of phytase production by solid substrate fermentation. *J. Ind. Microbiol. Biotechnol.* 30, 183-189. 10.1007/s10295-003-0027-3 12715256

[BIO047183C9] Chinedu S. N., Okochi V., Smith H. and Omidiji O. (2005). Isolation of cellulolytic micro-fungi involved in wood-waste decomposition: Prospects for enzymatic hydrolysis of cellulosic wastes. *Int. J. Biomed. Health. Sci.* 1, 0794-4748.

[BIO047183C11] Chuwech M., Rakariyatham N., Chandet N. and Tinoi J. (2016). Utilization of pretreated corn cobs for cellulase production by *Pycnoporus coccineus*. *KKU. Res. J.* 21, 310-318.

[BIO047183C12] Da Silva Delabona P., Lima D. J., Robl D., Rabelo S. C., Farinas C. S. and da Cruz Pradella J. G. (2016). Enhanced cellulase production by *Trichoderma harzianum* by cultivation on glycerol followed by induction on cellulosic substrates. *J. Ind. Microbiol. Biotechnol.* 43, 617-626. 10.1007/s10295-016-1744-8 26883662

[BIO047183C13] Dave B. R., Parmar P., Sudhir A., Singal N. and Subramanian R. B. (2014). Cellulases production under solid state fermentation using agro waste as a substrate and its application in saccharification by *Trametes hirsuta* NCIM. *J. Microbiol. Biotechnol. Food Sci.* 04, 203-208. 10.15414/jmbfs.2014-15.4.3.203-208

[BIO047183C14] de Oliveira S. L. D. R., Maciel T. C., de Oliveira Sancho S. and Rodrigues S. (2016). Solid-state production of cellulase by *Melanoporia* sp. CCT 7736: a new strain isolated from coconut shell (*Cocos nucifera* L.)*. Bioresour. Bioprocess.* 3, 9 10.1186/s40643-016-0086-8

[BIO047183C15] Deswal D., Khasa Y. P. and Kuhad R. C. (2011). Optimization of cellulase production by a brown rot fungus *Fomitopsis* sp. RCK2010 under solid state fermentation*. Bioresour. Technol.* 102, 6065-6072. 10.1016/j.biortech.2011.03.032 21470856

[BIO047183C16] Edgar R. C. (2004). MUSCLE: Multiple sequence alignment with high accuracy and high throughput. *Nucleic Acids Res.* 32, 1792-1797. 10.1093/nar/gkh340 15034147 PMC390337

[BIO047183C17] Felsenstein J. (1985). Phylogenies and the comparative method. *Am. Nat.* 125, 1-15. 10.1086/284325

[BIO047183C18] Fia G., Giovani G. and Rosi I. (2005). Study of β-glucosidase production by wine-related yeasts during alcoholic fermentation. a new rapid fluorimetric method to determine enzymatic activity. *J. Appl. Microbiol.* 99, 509-517. 10.1111/j.1365-2672.2005.02657.x 16108792

[BIO047183C20] Gautam S. P., Bundela P. S., Pandey A. K., Jamaluddin K., Awasthi M. K. and Sarsaiya S. (2011). Optimization for the production of cellulase enzyme from municipal solid waste residue by two novel cellulolytic fungi. *Biotechnol. Res. Int.* 2011, 1-8. 10.4061/2011/810425 PMC3042683 21350668

[BIO047183C21] Ginns J. (2017). Polypores of British Columbia (Fungi: Basidiomycota). *Tech. Rep.* 104, 1-256.

[BIO047183C23] Han X., Liu G., Song W., Qin Y. and Qu Y. (2017). Continuous feeding of spent ammonium sulphite liquor improves the production and saccharification performance of cellulase by *Penicillium oxalicum*. *Bioresour. Technol.* 245, 984-992. 10.1016/j.biortech.2017.09.042 28946207

[BIO047183C24] Haq I., Ali S., Javed M. M., Hameed U., Saleem A., Adnan F. and Qadeer M. A. (2010). Production of alpha amylase from a randomly induced mutant strain of *Bacillus amyloliquefaciens* and its application as a desizer in textile industry. *Pak. J. Bot.* 42, 473-484.

[BIO047183C25] Haque Z. U. and Mozaffar Z. (1992). Casein hydrolysate. I. Continuous production using enzyme bioreactors. *Top. Catal.* 5, 549-557. 10.1016/S0268-005X(09)80124-0

[BIO047183C26] Henrissat B. (1991). A classiﬁcation of glycosyl hydrolases based on amino acid sequence similarities. *Biochem. J.* 280, 309-316. 10.1042/bj2800309 1747104 PMC1130547

[BIO047183C27] Hesler L. R. and Smith A. H. (1979). North American species of Lactarius. Ann Arbor, Michigan: University of Michigan Library.

[BIO047183C28] Hillis D. M. and Bull J. J. (1993). An empirical test of bootstrapping as a method for assessing confidence in phylogenetic analysis. *Syst. Biol.* 42, 182-192. 10.1093/sysbio/42.2.182

[BIO047183C29] Isikgor F. H. and Becer C. R. (2015). Lignocellulosic biomass: a sustainable platform for the production of bio-based chemicals and polymers. *Polym. Chem.* 6, 4497-4559. 10.1039/C5PY00263J

[BIO047183C30] Jayachandran M., Xiao J. and Xu B. (2017). A critical review on health promoting benefits of edible mushrooms through gut microbiota. *Int. J. Mol. Sci.* 18, E1934 10.3390/ijms18091934 28885559 PMC5618583

[BIO047183C31] Justo A., Miettinen O., Floudas D., Ortiz-Santana B., Sjökvist E., Lindner D., Nakasone K., Niemelä T., Larsson K.-H., Ryvarden L. et al. (2017). A revised family-level classification of the Polyporales (Basidiomycota). *Fungal Biol.* 121, 798-824. 10.1016/j.funbio.2017.05.010 28800851

[BIO047183C32] Kalra M. K. and Sandhu D. K. (1986). Optimal production of cellulolytic enzymes and their location in *Trichoderma pseudokonigii*. *Biotechnol. Acta* 6, 161-166. 10.1002/abio.370060214

[BIO047183C33] Kirci H. (2006). *Pulp Industry Lecture Notes*. Trabzon, Turkey: Karadeniz Technical University, Forest Faculty Publication.

[BIO047183C34] Kondo M., Kita K. and Yokota H.-O. (2004). Feeding value to goats of whole-crop oat ensiled with green tea waste. *Anim. Feed Sci. Tech.* 113, 71-81. 10.1016/j.anifeedsci.2003.10.018

[BIO047183C35] Krueger D. (2002). Monographic studies in the genus polyporus (Basidiomycotina). *PhD thesis*, University of Tennessee, Knoxville, TN, USA.

[BIO047183C36] Krüger D. and Gargas A. (2004). The basidiomycete genus *Polyporus* – an emendation based on phylogeny and putative secondary structure of ribosomal RNA molecules. *Feddes Repert.* 115, 530-546. 10.1002/fedr.200311052

[BIO047183C38] Kumaran S., Sastry C. A. and Vikineswary S. (1997). Laccase, cellulase and xylanase activities during growth of*Pleurotus sajor-caju* on sagohampas. *World J. Microbiol. Biotechnol.* 13, 43-49. 10.1007/BF02770806

[BIO047183C40] Lamb J. and Loy T. (2005). Seeing red: the use of Congo Red dye to identify cooked and damaged starch grains in archaeological residues. *J. Archaeol. Sci.* 32, 1433-1440. 10.1016/j.jas.2005.03.020

[BIO047183C41] Li Q., Yi L., Marek P. and Iverson B. L. (2013). Commercial proteases: present and future. *FEBS Lett.* 587, 1155-1163. 10.1016/j.febslet.2012.12.019 23318711

[BIO047183C42] Mao J., Nong X., Yang Y. and Liang Z. (2015). *Trichoderma reesei Utilized Tea Residue to Production Cellulase*. Atlantis Press.

[BIO047183C44] Miller G. L. (1959). Use of dinitrosalicylic acid reagent for determination of reducing sugar. *Anal. Chem.* 31, 426-428. 10.1021/ac60147a030

[BIO047183C45] Montoya S., Sanchez O. J. and Levin L. (2015). Production of lignocellulolytic enzymes from three white-rot fungi by solid-state fermentation and mathematical modeling. *Afr. J. Biotechnol.* 14, 1304-1317. 10.5897/AJB2014.14331

[BIO047183C46] Onofre S. B., Santos Z. M. Q., Kagimura F. Y. and Mattiello S. P. (2015). Cellulases produced by the endophytic fungus *Pycnoporus sanguineus* (L.) Murrill. *Afr. J. Agric. Res.* 10, 1557-1564. 10.5897/AJAR2015.9487

[BIO047183C47] Prakasham R. S., Subba C. S. and Sarma P. N. (2006). Green gram husk—an inexpensive substrate for alkaline protease production by *Bacillus* sp. in solid-state fermentation*. Bioresour. Technol.* 97, 1449-1454. 10.1016/j.biortech.2005.07.015 16140528

[BIO047183C48] Rajendran R., Radhai R., Karthik S. S. and Rajalakshmi V. (2015). Utilization of cellulosic biomass as a substrate for the production of bioethanol. *Int. J. Environ. Sci. Technol.* 5, 743-753.

[BIO047183C49] Ramachandran S., Patel A. K., Nampoothiri K. M., Francis F., Nagy V., Szakacs G. and Pandey A. (2004). Coconut oil cake—a potential raw material for the production of α-amylase. *Bioresour. Technol.* 93, 169-174. 10.1016/j.biortech.2003.10.021 15051078

[BIO047183C50] Ramapraba A. (2004). Effect of media and environmental factors on growth and cellulase production by *Microporus xanthopus*. Bachelor thesis, Universiti Malaysia, Sarawak, Kota Samarahan, Sarawak, Malaysia.

[BIO047183C51] Rana S. and Kaur M. (2012). Isolation and screening of cellulase producing microorganisms from degraded wood. *Int. J. Pharm. Biol. Sci.* 2, 10-15.

[BIO047183C52] Ravindran R. and Jaiswal A. K. (2016). Microbial enzyme production using lignocellulosic food industry wastes as feedstock: a review. *Bioengineering* 3, 30 10.3390/bioengineering3040030 PMC5597273 28952592

[BIO047183C53] Ronquist F., Teslenko M., van der Mark P., Ayres D. L., Darling A., Höhna S., Larget B., Liu L., Suchard M. A. and Huelsenbeck J. P. (2012). Mrbayes 3.2: Efficient bayesian phylogenetic inference and model choice across a large model space. *Syst. Biol.* 61, 539-542. 10.1093/sysbio/sys029 22357727 PMC3329765

[BIO047183C54] Sadhu S. and Maiti T. K. (2013). Cellulase production by bacteria: A Review. *Br. Microbiol. Res. J.* 3, 235-258. 10.9734/BMRJ/2013/2367

[BIO047183C57] Shahzadi T., Anwar T., Iqbal Z., Anjum A., Aqil T., Bakhtawar, Afzal A., Kamran M., Mehmood S. and Irshad M. (2014). Induced production of exoglucanase, and β-glucosidase from fungal co-culture of *T. viride* and *G. lucidum*. *Adv. Biosci. Biotechnol.* 5, 426-433. 10.4236/abb.2014.55051

[BIO047183C58] Sherief A. A., El-Naggar N. E.-A. and Hamza S. S. (2010). Bioprocessing of lignocellulosic biomass for production of bioethanol using thermotolerant *Aspergillus fumigatus* under solid state fermentation conditions. *Biotechnol. J.* 9, 513-522. 10.3923/biotech.2010.513.522

[BIO047183C59] Sodhi H. K., Sharma K., Gupta J. K. and Soni S. K. (2005). Production of a thermostable α-amylase from *Bacillus* sp. PS-7 by solid state fermentation and its synergistic use in the hydrolysis of malt starch for alcohol production. *Proc. Biochem.* 40, 525-534. 10.1016/j.procbio.2003.10.008

[BIO047183C60] Stamatakis A. (2006). RAxML-VI-HPC: Maximum likelihood-based phylogenetic analyses with thousands of taxa and mixed models. *Bioinformatics* 22, 2688-2690. 10.1093/bioinformatics/btl446 16928733

[BIO047183C61] Sukumaran R. K., Singhania R. R. and Pandey A. (2005). Microbial cellulases-production, applications and challenges. *J. Sci. Ind. Res. India* 64, 832-844.

[BIO047183C62] Tao H., Bausch C., Richmond C., Blattner F. R. and Conway T. (1999). Functional genomics: Expression analysis of *Escherichia* *coli* growing on minimal and rich media. *J. Bacteriol.* 181, 6425-6440. 10515934 10.1128/jb.181.20.6425-6440.1999 PMC103779

[BIO047183C63] Teixeira Da Silva V. D. C., De Souza Coto A. L., De Carvalho Souza R., Neves M. B. S., Gomes E. and Bonilla-Rodriguez G. O. (2016). Effect of pH, temperature, and chemicals on the endoglucanases and β-glucosidases from the thermophilic fungus *Myceliophthora heterothallica* F.2.1.4. Obtained by solid-state and submerged cultivation. *Biochem. Res. Int.* 2016, 9781216 10.1155/2016/9781216 27242927 PMC4875970

[BIO047183C64] Tutus A., Cicekler M., Ozdemir F. and Yilmaz U. (2014). Evaluation of *Diospyros kaki* grown in Kahramanmaraş in pulp and paper production. National Mediterranean Forest and Environment Symposium, Isparta, Turkey, 775-784.

[BIO047183C66] Vintila T., Dragomirescu M., Strava S. and Croitoriu V. (2009). Enzymatic hydrolysis of agricultural lignocellulosic biomass. *Zooteh. Si Biotehnol.* 42, 1-5.

[BIO047183C67] Vu D., Groenewald M., de Vries M., Gehrmann T., Stielow B., Eberhardt U., Al-Hatmi A., Groenewald J. Z., Cardinali G., Houbraken J. et al. (2019). Large-scale generation and analysis of filamentous fungal DNA barcodes boosts coverage for kingdom fungi and reveals thresholds for fungal species and higher taxon delimitation. *Stud. Mycol.* 92, 135-154. 10.1016/j.simyco.2018.05.001 29955203 PMC6020082

[BIO047183C69] Yang D., Liang J., Wang Y., Sun F., Tao H., Xu Q., Zhang L., Zhang Z., Ho C.-T. and Wan X. (2015). Tea waste: an effective and economic substrate for oyster mushroom cultivation. *J. Sci. Food Agric.* 96, 680-684. 10.1002/jsfa.7140 25690537

[BIO047183C70] Zecarias G., Duangporn P. and Siripong P. (2016). Screening of fungi producing ligninolytic enzymes by plate test technique. *KKU. Res. J.* 22, 200-209.

[BIO047183C71] Zhang Y. H.-P. and Lynd L. R. (2006). A functionally based model for hydrolysis of cellulose by fungal cellulase. *Biotechnol. Bioeng.* 94, 888-898. 10.1002/bit.20906 16685742

[BIO047183C72] Zhou J.-L., Zhu L., Chen H. and Cui B.-K. (2016). Taxonomy and phylogeny of polyporus group melanopus (Polyporales, Basidiomycota) from China. *PLoS ONE* 11, e0159495 10.1371/journal.pone.0159495 27486931 PMC4972403

